# Effects of Electromagnetic Fields on the Microstructure of Laser Cladding

**DOI:** 10.3390/ma15124198

**Published:** 2022-06-13

**Authors:** Yongjun Shi, Xiaoyu Zhou, Xiaogang Wang, Xingteng Feng, Laida Peng

**Affiliations:** College of Electromechanical Engineering, China University of Petroleum (East China), Qingdao 266580, China; zxy_013@163.com (X.Z.); wxgang6688@163.com (X.W.); z20040055@s.upc.edu.cn (X.F.); upcpengld@163.com (L.P.)

**Keywords:** laser cladding, electromagnetic assistance, microstructure

## Abstract

The fast heating and quenching of laser cladding increase the internal stresses in the cladding layer. Moreover, the quick condensation of the molten pool leads to an uneven distribution of the internal elements and coarse grains of the structure. To address the above defects and increase the molding quality of laser cladding, an electromagnetic field was introduced into the laser cladding technique, and the effects of the external assisted electromagnetic field on the mixed metal fluid in the molten pool were explored. On this basis, the action of the electromagnetic field on the flow states of the molten pool was further analyzed. The results demonstrate that after introducing electromagnetic assistance, the material flow in the molten pool accelerated as a response to the periodic changes in electromagnetic forces and the influences of the electromagnetic field on crystallization, thus refining the grains and improving the grain distribution uniformity in the cladding layer. The dendritic crystals in the cladding layer decreased, while the isometric crystals and the cellular-like dendrites increased. The element distribution in the cladding layer increased in uniformity. Additionally, this method can decrease the dilution rate of the cladding layer and improve its overall hardness. A laser-cladding test of the Ni-based powder was carried out on the AISI 1045 steel surface under the coaxial powder-feeding mode. Moreover, the influences of the electromagnetic field on the microstructure of the laser-cladding layer were compared, and the causes of the changes were disclosed.

## 1. Introduction

Laser cladding is a surface-strengthening technology that quickly applies a high-performance coating to the workpiece surface by using high-energy laser beams. This technology can apply a high-performance coating to core equipment surfaces with a few high-quality alloy materials, thus prolonging the service life of the equipment. Laser cladding has been extensively applied in the petroleum, transportation, and aerospace industries [1,2,3].

Nevertheless, various defects, like cracks and pores, are produced on the cladding layer, due to differences in thermophysical properties between the base and cladding layer materials and the fast heating and cooling of the molten pool during the cladding process. These defects limit the application of a cladding layer in industrial production [4,5,6]. Given the fast heating and cooling of laser cladding, the internal stress of the cladding layer is stronger than the yield strength, thus producing cracks. Melted metals contain gases that cannot escape in a timely manner during fast condensation, thus resulting in pores in the cladding layer. Moreover, the stress concentrations at the tips of the pores cause cracks.

To address the above defects and improve the molding quality of laser cladding, research both at home and abroad in this field has explored various approaches. The major research directions include improving the cladding process by establishing the optimal technological parameters, increasing the wettability by adjusting the element contents of the cladding layer, and applying an external force field during cladding. Abioye et al. [7,8] applied an Inconel 625 alloy coating to a 304 stainless-steel surface by using laser wire-feeding cladding technology and analyzed the molding quality, dilution rate, and corrosion resistance of the coating using different technological parameters. According to their analysis, the Inconel 625 coating applied by laser cladding achieved a relatively good corrosion resistance when the Fe content was low (the dilution rate was low). Fesharaki et al. [9] applied an Inconel 625 cladding layer to an Inconel 738 alloy surface by TIG welding and observed the sections of the samples. They observed the formation of many cracks at the boundary between the base and the coating. The sensitivities to cracks of the base and coating materials increased due to the high heat input. Silwal et al. [10] studied an Inconel 625 coating prepared by hot-wire-assisted arc cladding. Under a relatively high current intensity, the coating-based interface also developed many cracks. Wang et al. [11] paved the cladding material onto a base surface and the prepared Ni-based cladding layer. In the experiment, a large-area cladding layer was obtained through multi-channel joining; a high jointing rate could decrease the sensitivity of the entire cladding layer to cracks while ensuring its flatness.

To increase the uniformity of the distribution of elements and microstructure in the depth direction of the molten pool, researchers have attempted to introduce external assistance to the molding process of laser cladding, thus increasing the mobility of the molten pool and achieving important breakthroughs. Ram et al. [12] strengthened the mobility of liquid metals in the molten pool through a pulse current during the arc welding of Inconel 718 alloy and facilitated the breakage of the solid–liquid interface in the molten pool along the dendrites before condensation, thus forming a refined microstructure. Liu et al. [13] studied the precipitation state of the Laves phase in a GH4619 alloy molded through laser cladding, with the assistance of electromagnetic stirring. Their results show that the electromagnetic stirring facilitated the flow of liquid metals in the molten pool, and the Laves phase changed from networked precipitation into fine particles. The Nb content in the dendrite trunks increased effectively, thus increasing the microhardness of the molding samples. Meng et al. [14] introduced an alternating field to laser deep-welding and compared the flow behaviors of metals in the welding molten pool with and without alternating magnetic stirring, using calculations. They found that the electromagnetic field intensified the flow in the molten pool and facilitated the refinement of the weld’s metal crystals.

Recently, electromagnetic-assisted laser cladding has been studied extensively. Some progress in the numerical simulation of laser cladding, the role of the magnetic field on the molten pool, and the microstructure of the cladding layer under magnetic-field assistance has been achieved. However, intensive studies on the preparation of excellent laser cladding layers under electromagnetic assistance and the analysis of the microstructure of the laser cladding layer are needed. In this study, a coaxial powder-feeding laser-cladding experiment was performed under electromagnetic assistance, using AISI 1045 steel as the base and a Ni-based self-melting metal powder as the cladding material. The influencing mechanism of the electromagnetic field on laser cladding was intensively investigated by observing the microstructure of the specimens, the element distribution, and the micro-hardness of the cladding layer.

## 2. Experimental Method

### 2.1. Materials

AISI 1045 steel was selected as the base material and self-melting Ni60AA metal powder was used as the cladding material, which has a low melting point and good self-melting properties. In addition, Ni60AA has excellent wear, heat, and oxidation resistance and is widely used in additive manufacturing. The chemical compositions of the base and cladding materials are shown in Table 1 and Table 2, respectively.

**Figure 1:**
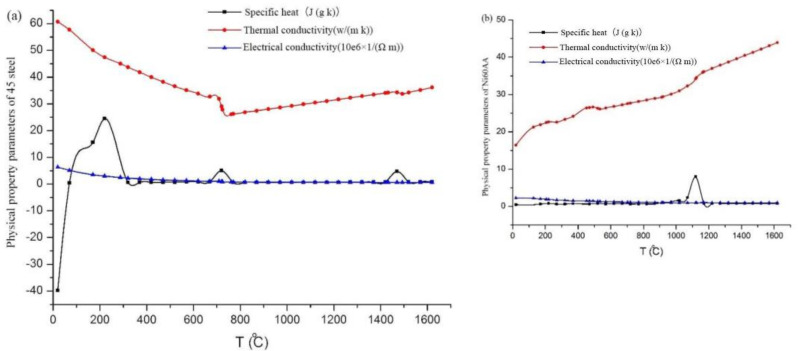
Changes in thermophysical properties with temperature: (**a**) AISI 1045 steel; (**b**) Ni60AA.

During cladding molding, the thermophysical properties of the base and cladding materials are related to temperature. Hence, the variation trends of the thermophysical properties (e.g., specific heat, thermal conductivity, and electric conductivity) of the base and cladding materials with temperature are presented in Figure 1.

### 2.2. Experimental Platform

**Figure 2:**
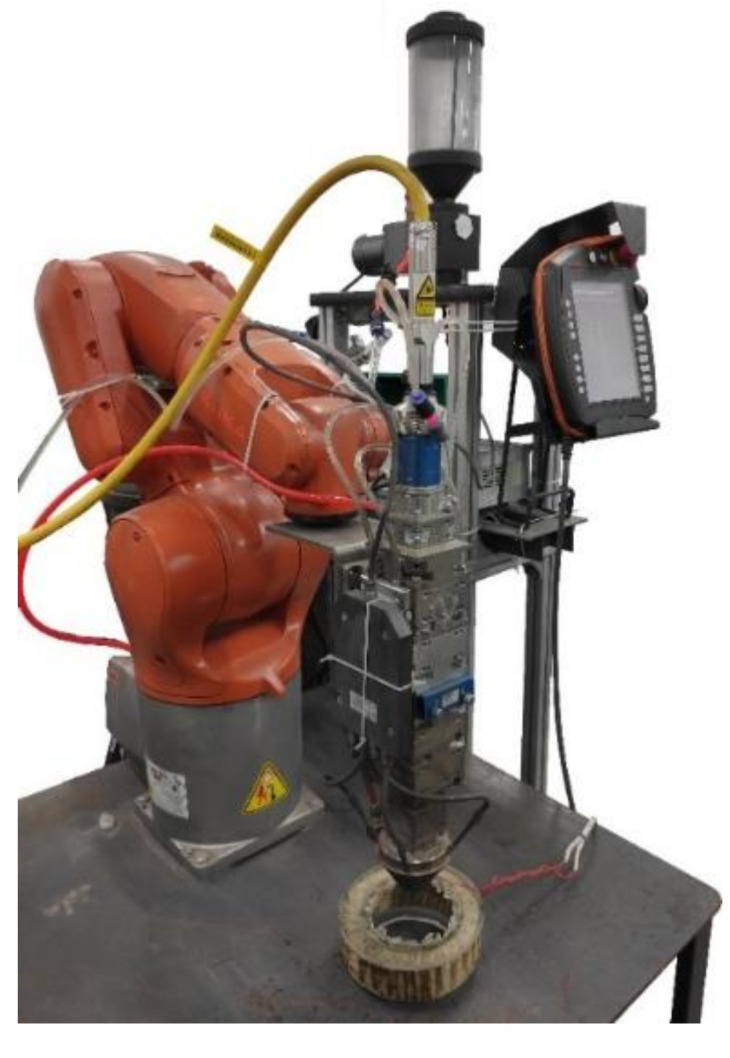
Experimental platform.

A complete experimental platform is composed of six systems: the laser generation and transmission system, the metal powder-transmitting system, the shielding gas transmission system, and the magnet exciting coil, cooling, and motion-control systems. The laser generates a high-energy laser beam for the heat source, while the metal powder conveyor (IGS-3J, Henan Coal Research Institute wear-resisting Technology Co., Ltd., Henan, China) transfers the metal powder to the coaxial powder feeder, which then forms a molten pool under the action of the high-energy laser and results in metallurgical bonding with the matrix. The shielding gas system can prevent the oxidation of materials in a high-temperature environment and provides air pressure for the powder feeder, to ensure the normal transportation of the metal powder. The magnet exciting coil system applies electromagnetic force to the molten pool formed during laser processing. The cooling system is composed of a cooling machine and a cooling pipeline. The cooling system continuously circulates the distilled water of the cooling line built into the laser cladding head and cools the cooling water for the rising temperature again, to ensure that the cladding head is within an appropriate temperature range. The motion control system consists of a mechanical arm (i.e., KUKA), which can complete various forms of machining paths. The physical experimental platform is shown in Figure 2. All systems are interconnected and comprise the coaxial powder-feeding laser cladding mechanism under electromagnetic assistance.

The alternating magnetic field produced in the coils during the experiment was measured with an HT201 Gauss meter.

### 2.3. Selection of Technological Parameters

In coaxial powder-feeding laser cladding technology, effective technological parameters can not only ensure bonding stability between the cladding layer and the base materials during laser cladding but also form a relatively stable morphology, satisfying the continuity and size stability needs of the cladding layer and avoiding large-area processing defects on the specimens.

In this research, the laser power (*P*), the processing velocity (*v_0_*), the powder-feeding amount (*L*), and the defocusing amount (*d_1_*) were viewed as the major factors that influence laser cladding. In the experiment, the protective air pressure (*p_b_*) and the powder-feeding pressure (*p_f_*) were constant: *p_b_* = 0.2 MPa and *p_f_* = 0.1 MPa. The diameter of the laser spot was 3 mm.

To determine the order of significance of the above technological parameters in terms of molding and to recognize their appropriate intervals, the above parameters were optimized through an orthogonal test. The test parameters are shown in Table 3. Each parameter has five values. The cladding layer was evaluated comprehensively, according to its size, size stability, defect quantity, and bonding strength with the base. Moreover, the orthogonal test results were analyzed.

A qualitative method was used for scoring, and a benchmark of 80 points was added to or subtracted from each sample. The main evaluation indexes were the combination, the size, and the number of defects. The binding situation is divided into three aspects: the presence/absence of binding, the depth of the transition zone, and the width of the heat-affected zone. For a sample that fails to combine, the fraction will be subtracted; for a sample with an extremely deep or shallow transition zone, the fraction will be subtracted; for a sample with a high combination degree and a moderate transition zone, a set fraction will be added. In terms of size, the score will be low if the cladding layer is extremely large or small. In terms of cladding defects, points will be subtracted from the score when excessive cracks, uneven surfaces, and pores occur. The highest score was 135, while the lowest score was 25.

**Figure 3:**
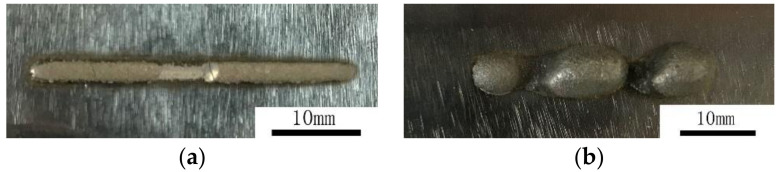

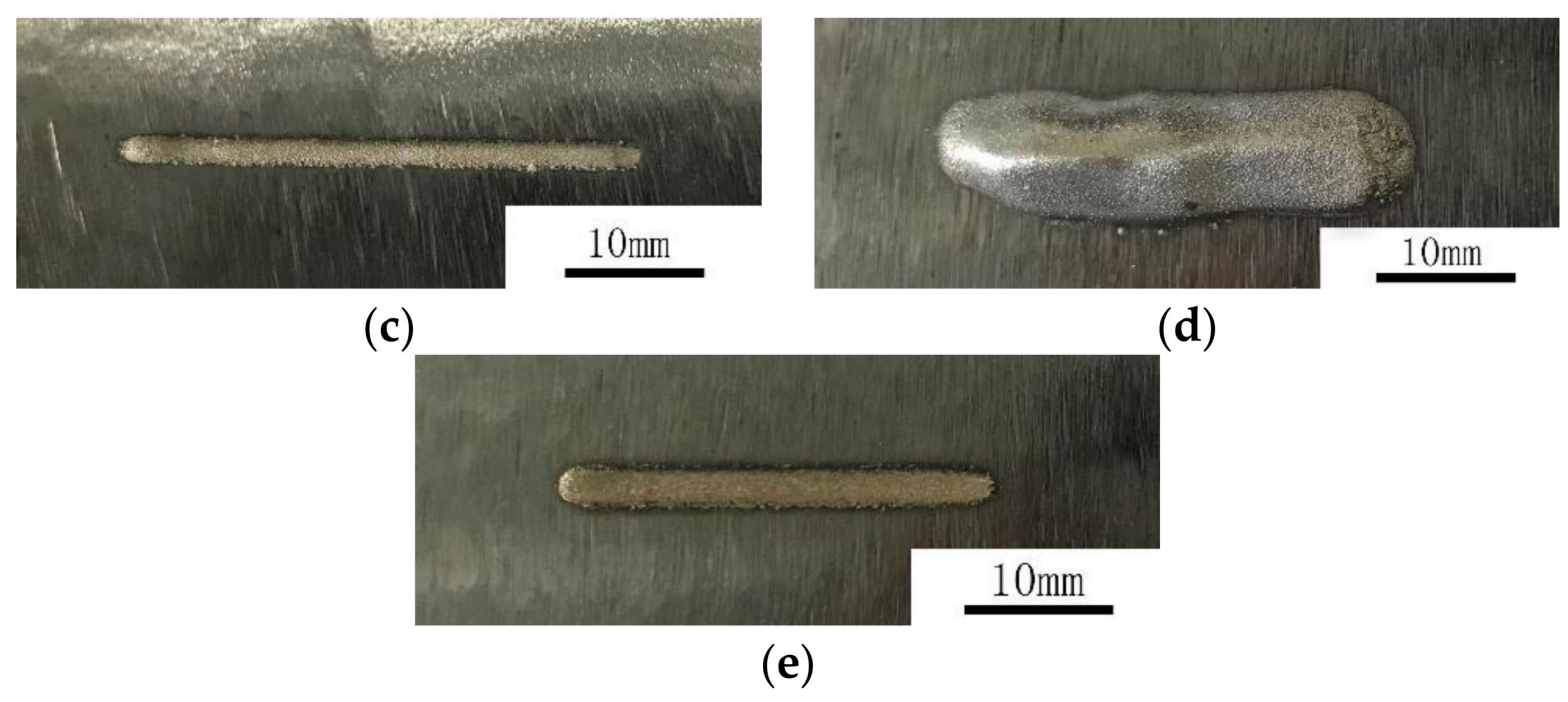
Morphology of specimens in the orthogonal test. (**a**) No. 3; (**b**) No. 6; (**c**) No. 9; (**d**) No. 21; (**e**) No. 25.

Each specimen was qualitatively scored after the above orthogonal test. The major evaluation indexes include the binding state, the size, and the defect quantity. All species were scored, using a score of 80 as the benchmark. The binding state was divided into the binding existence, the depth of the transition area, and the width of the heat-affected zone (HAZ). With respect to size, the score of the cladding layer decreased when the size was inadequate or extremely large or small. With respect to the defects of the cladding layer, the scores decreased when excessive cracks, uneven surfaces, and pores existed. The representative specimen morphologies are presented in Figure 3.

Specimen #3 presents low binding strength and many cracks on the cladding layer, but its size is relatively stable. Specimen #6 experienced a discontinuous cladding process and metal powder accumulation during the test; the cladding layer is coarse and the binding strength is low. Specimen #9 has a relatively narrow cladding layer and a superficial transition area. Specimen #21 failed to bind with the cladding layer, which is extremely wide. Moreover, the cladding layer experienced red-hot due to powder accumulation in the processing. Hence, Specimen #21 obtained the lowest scores and the poorest technological parameters. Specimen #25 exhibited good performance in terms of morphology and binding strength and has very few defects in the cladding layer. This specimen obtained the highest scores and has relatively good technological parameters. The maximum and minimum scores are 135 and 25, respectively.

The optimal intervals of the molding technological parameters were *P* > 1200 W, *v_0_* = 5–8 mm/s, and *d_1_* = 12–18 mm. A relatively low powder-feeding amount can meet the molding quality requirements. In this study, *L* was fixed at 1.18 g/min in the laser cladding test under electromagnetic assistance. The magnet exciting coil used 1000 turns of a copper conductor with a diameter of 0.8 mm. An alternating voltage of 220 V 50 Hz was applied.

### 2.4. Test Method of Molding Parts

Specimens with representative significance were selected for metallographic analysis. The length of the metallographic specimens was 10 mm. The microstructures of the specimens were analyzed by combining metallographic light microscopy and scanning electron microscopy. The binding zone between the cladding layer and the base was selected for EDS testing. The hardness of the cladding layer was tested using a microhardness meter and the applied load of the instrument was 200 g, which was maintained for 5 s. The mid-line of the specimens was used as the position of the hardness testing point. Six and five testing points were chosen at intervals of 0.2 mm on the cladding layer and the base, respectively. The bottom testing point, which was nearest to the cladding layer, was attached to the binding line in the test.

## 3. Results and Analysis

### 3.1. Effects of Electromagnetic Field Assistance on the Macroscopic Morphology of Specimens

To explore the influencing mechanism of the external electromagnetic field in the laser cladding process and its influence on the final molding morphology, the effects of the alternating electromagnetic field on the microscopic morphology of the molten pool and the cladding layer were further analyzed, based on the optimization of the above technological parameters. In the experiment, some parameters were fixed, including *p_b_* = 0.2 MPa, *p_f_* = 0.1 Mpa, and the laser pot diameter = 3 mm. The controller voltage of the powder feeder was 5 V (the corresponding *L* = 1.18 g/min). The AISI 1045 steel and the Ni60AA self-melting powder were used as the base and cladding layer materials, respectively. The technological parameters are shown in Table 4.

**Figure 4:**
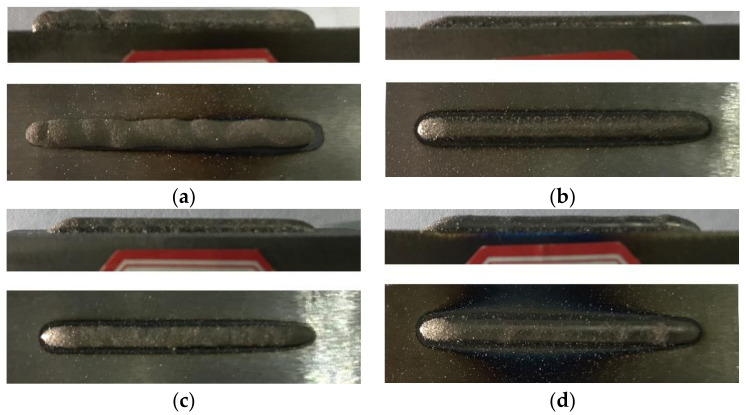
Macroscopic morphology of the specimens. (**a**) No. 1; (**b**) No. 2; (**c**) No. 3; (**d**) No. 4.

After experimental studies based on the technological parameters in Table 4, the macroscopic morphologies of the molding specimens were analyzed. The results show that after optimizing the technological parameters, the macroscopic morphologies of the specimens improved significantly, basically causing no cladding layer defects such as inadequate or excessive binding strength. The macroscopic morphologies of the specimens are shown in Figure 4.

Specimens #1 and #2 are cladding layers without electromagnetic field assistance, while Specimens #3 and #4 are cladding layers with electromagnetic field assistance. The surface variations of Specimens #3 and #4 were flatter and the quality of binding with the bases was smoother than those of Specimens #1 and #2.

### 3.2. Effects of Electromagnetic Field on the Microstructure

**Figure 5:**
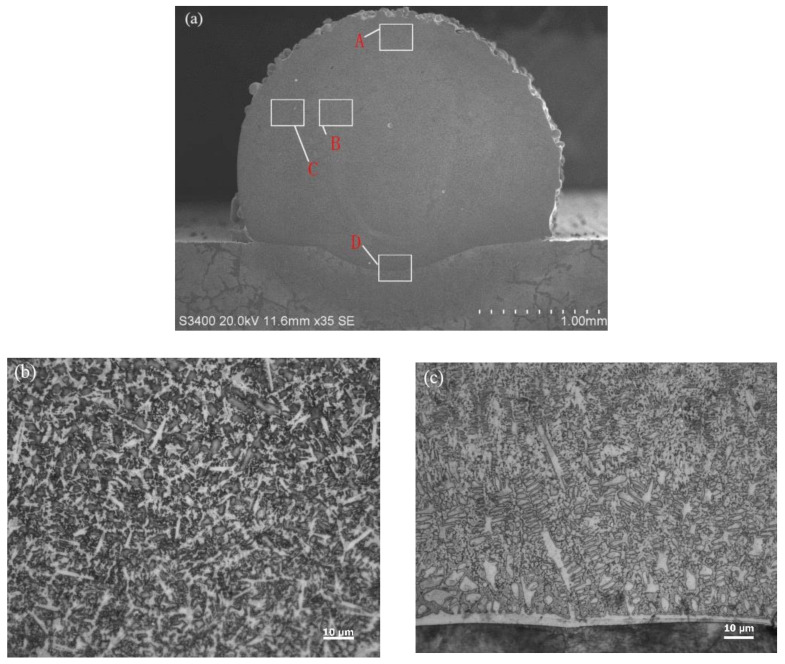
Structures of specimens without electromagnetic field assistance. (**a**) The overall morphology of the sample; (**b**) microstructure at point A; (**c**) microstructure at point D.

The external electromagnetic field has positive effects on the macroscopic morphology of the cladding layer. Therefore, the influence of the electromagnetic field on the microstructure of specimens requires further exploration to reveal the effects of the electromagnetic field on the performance of the cladding layer. Figure 5 shows the sample structure without electromagnetic field assistance. The molding technological parameters are listed in Table 5. The positions of the characteristic points of the cladding layer are shown in Figure 5a. The microstructures of the top middle position (point A) and bottom middle position (point D) of the cladding layer under an amplification of 1000 are shown in Figure 5b,c. For the bottom structures of the cladding layer, stripped crystals, dendrites, isometric crystals, and cellular-like crystals were developed from the lower base to the upper positions. Among them, dendrites and cellular-like crystals were developed in the mixture. The lower positions had more dendrites than cellular-like crystals. The upper position had more cellular-like crystals than dendrites.

At the bottom of the cladding layer, stripped crystals were distributed in the entire transition area between the cladding layer and the base. Stripped crystals are formed by the processing characteristics of coaxial powder-feeding laser cladding. The laser penetrated through the powder beams and continued to irradiate onto the upper surfaces of the specimens. After energy loss, the laser energy absorbed by the base can heat the upper surface of the base instantly. After the metal liquid was formed by the melting of Ni60AA powder, which is rich in Ni elements, and dropped onto the upper surface of the base, the Ni60AA came in contact with AISI 1045 steel under a high temperature, during which process their chemical elements diffused mutually. After the laser heat source passed through, the temperature dropped, and the diffused elements were condensed in the transition area accordingly. Fe, which diffused upward in the base, formed an Fe-containing alloy phase in the cladding layer. The bond zone, which was closest to the cladding layer and the base, was rich in Fe, thus forming large-sized stripped crystals. The stripped crystals condensed first in the molten pool in the same section.

**Figure 6:**
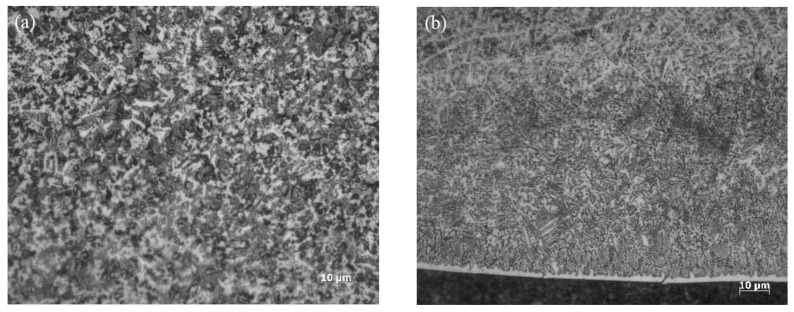
Sample structure with electromagnetic field assistance. (**a**) Microstructure at point A of Figure 5a; (**b**) microstructure at point D of Figure 5a.

Figure 6 shows the sample structure with electromagnetic field assistance. The technological parameters are the same as those in Table 5, except for the addition of the electromagnetic field assistance. The microstructures at points A and D of the cladding layer under an amplification of 1000 are shown in Figure 6a,b, respectively. Points A and D are shown in their corresponding positions in Figure 5a.

According to the analysis, many isometric crystals and a few cellular-like crystals were produced in the central region at the top of the cladding layer. The dendrites broke in the cladding process due to the stirring effect of the external magnetic field. The broken dendrite arms were carried along to other positions in the molten pool with the metal fluid and continued the crystallization process as a new nucleation. All parts of the broken dendrites formed small-sized dendrites at the top of the cladding layer. Some of these dendrites grew continuously, while others formed cellular-like crystals and isometric crystals. As the temperature of the molten pool dropped, the metals condensed gradually. The quantity of small-sized isometric and cellular-like crystals was higher than that of dendrites, thus realizing the crystal refining effect. Figure 6b shows that the number of dendrites connected to the stripped crystals at the bottom of the cladding layer decreased significantly, and the dendrites at the bottom of the molten pool were pulled away from the cladding layer by the accelerated flow under electromagnetic force and continued to grow.

**Figure 7:**
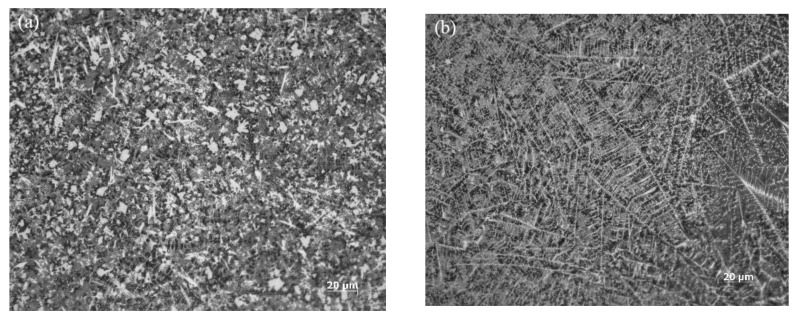
Sample structure with electromagnetic field assistance. (**a**) Microstructure at point C of Figure 5a; (**b**) microstructure at point B of Figure 5a.

The microstructures at the center of the cladding layer under electromagnetic field assistance are shown in Figure 7. The microstructure at the edges (point C in Figure 5a) of the center of the cladding layer is shown in Figure 7a. The microstructure near the central axis (point B in Figure 5a) in the center of the cladding layer is shown in Figure 7b.

**Figure 8:**
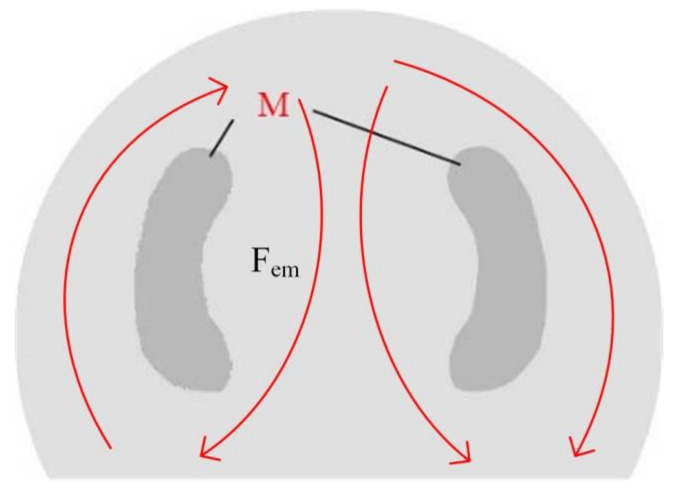
Dendritic distribution region.

According to our analysis, the microstructure at point C was basically the same as that at the top of the cladding layer, showing many isometric crystals and small dendrites, which were accompanied by a few cellular-like crystals. The lateral region near the central axis has many small dendrites but very few isometric crystals. These dendrites are distributed symmetrically at the left and right of the cladding layer, forming the M region shown in Figure 8.

The flow morphology in the molten pool is related to the internal driving forces in the molten pool, including the electromagnetic, Marangoni, and buoyancy forces. Electromagnetic force plays the dominant role [15,16]. The flow behaviors of melting metals along the external edges, mid-line, and bottom of the cladding layer under the action of electromagnetic force in the molten pool are shown in Figure 8. The flows at the left and right of the molten pool are opposite. The dendrites at the bottom of the molten pool broke and moved upward in the cladding layer due to the application of electromagnetic force. The metal flow in the molten pool increased the dendrites in zone M of the cladding layer. The broken dendrites, which continued to grow, the isometric crystals, and the cellular-like crystals were all influenced by the acceleration of the metal flows. The crystals on the stressed flowing route of the melted metals were easy to break. However, the metal flow in the central zone of the left and right looped routes (zone M) was relatively weak. Therefore, many large-sized dendrites were concentrated in zone M.

### 3.3. Effects of the Electromagnetic Field on Phases in the Molten Pool

**Figure 9:**
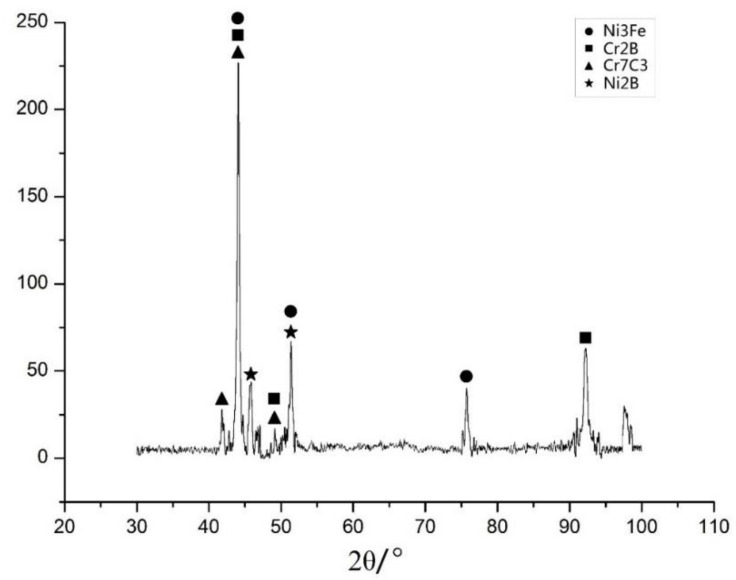
XRD atlas.

A 3-millimeter wide plane was polished at the upper part of the cladding layer for X-ray diffraction (XRD) analysis. A copper target was used, with angles ranging from 30° to 100° at a speed of 4°/min. Figure 9 shows the XRD pattern of the sample assisted by an electromagnetic field, and Table 6 shows the process parameters.

According to the XRD spectral analysis of the specimens, the cladding layer mainly contained Ni_3_Fe, Cr_2_B, Cr_7_C_3_, Ni_2_B, and a certain amount of iron–carbon compounds. Moreover, most iron–carbon compounds in the cladding layer appeared in the form of martensite because C formed an oversaturated solid solution in the *α*-Fe. The metal compound Ni_3_Fe content in the cladding layer is far higher than the other components. Ni_3_Fe can increase the overall tenacity of materials. Moreover, the cladding layer contains other types of Fe-Ni solid solution, which is precipitated first at the condensation of the molten pool. Additionally, the high Fe, Cr, Ni, B, and C contents form compounds. The XRD spectra of the specimens with and without electromagnetic field assistance are basically the same, indicating that electromagnetic assistance had a relatively small influence on the material phases in the laser cladding. The temperature field distribution in the molten pool during cladding is the major factor that influenced the phases in the cladding layer.

### 3.4. Effects of the Electromagnetic Field on Microhardness

**Figure 10:**
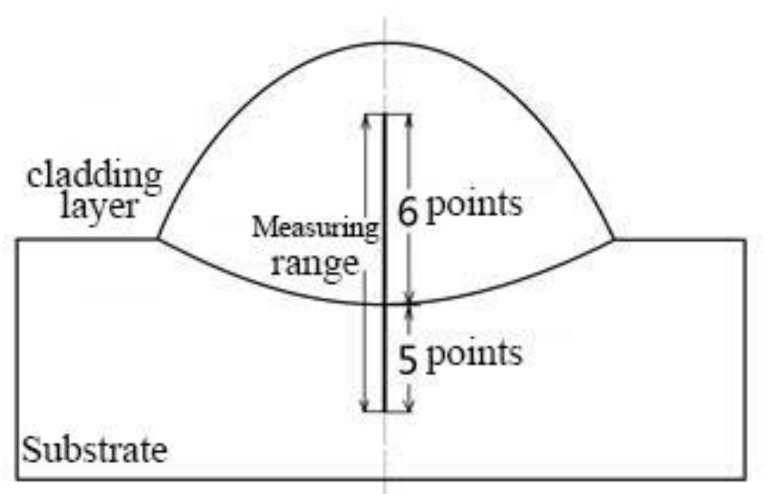
Schematic illustration of micro-hardness test points.

The applicable working conditions of the metal materials are closely related to the microhardness of the materials. The hardness of the materials can be tested using many methods. The mid-line of the specimens was selected to set the hardness testing points. Six and five testing points were set at intervals of 0.2 mm on the cladding layer and the base, respectively. The bottom testing point nearest the cladding layer was attached to the binding line in the test. The microhardness test applied a load of 200 g for 5 s. The microhardness test method is shown in Figure 10.

**Figure 11:**
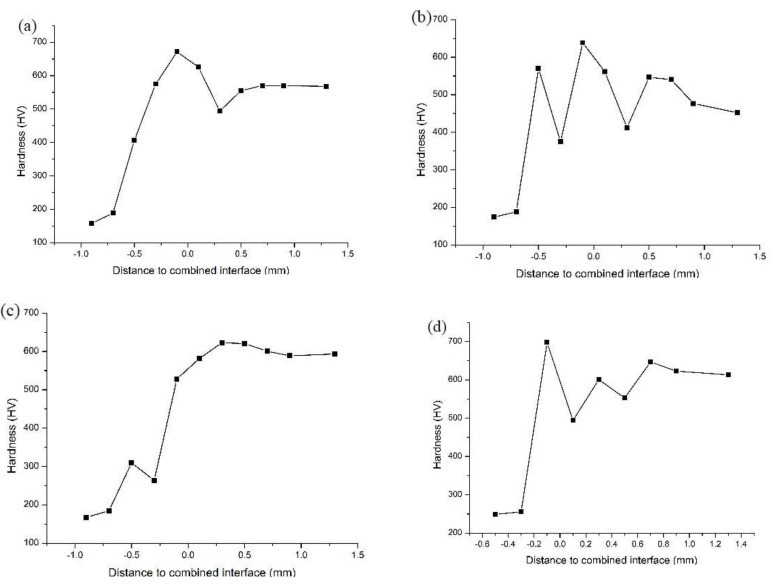
Trend of Vickers hardness. (**a**) Sample 1; (**b**) sample 2; (**c**) sample 3; (**d**) sample 4.

The microhardness measuring results of the laser cladding specimens are shown in Figure 11. According to our analysis, the Vickers hardness of the base ranged from 100 HV to 200 HV. The hardness value of the cladding layer covers a relatively large span due to the differences in technological parameters, ranging from 500 HV to 700 HV. The technological parameters are shown in Table 4.

The Vickers hardness values of Specimens #1 and #2 without electromagnetic field assistance are shown in Figure 11a,b. Clearly, the microhardness of Specimens #1 and #2 increased suddenly in the transition area and then remained stable in the cladding layer area. The hardness of Specimen #1 in the cladding layer was basically maintained at approximately 550 HV. The hardness of Specimen #2 fluctuated greatly around 500 HV because of the uneven distribution of the Fe diffused from the base in the cladding layer. The Vickers hardness values of Specimens #3 and #4 with electromagnetic field assistance are shown in Figure 11c,d. With respect to the variation trend, their microhardness from the base to the cladding layer increased quickly in the beginning, then decreased, to some extent, and finally, tended to be stable.

In terms of element distribution in Specimens #3 and #4 with electromagnetic field assistance, the “platform” in the transition area of the Fe element moved along the cladding layer, while the excessive concentration of the base elements in the cladding layer decreased the hardness. Therefore, the variation trend of hardness is consistent with the element distribution in Specimens #3 and #4. The hardness of Specimens #3 and #4 was stable at approximately 600 HV, which is slightly higher than that for Specimens #1 and #2. This phenomenon occurred because the elements in the molten pool, which were influenced by the external electromagnetic field, were mixed more evenly in the cladding process, thus improving the overall microhardness of the cladding layer.

## 4. Conclusions

The coaxial feeding laser cladding of Ni-base powder on the AISI 1045 steel surface when assisted by an electromagnetic field was studied according to the defects of the cladding layer using the existing laser-cladding technology, such as excessive internal stress, the uneven distribution of elements in the molten pool, and the coarseness of the grain structure.

The influence of the electromagnetic field on coaxial powder-feeding laser cladding was investigated. After optimizing the molding technological parameters, the influence of the electromagnetic field on macroscopic morphologies, microstructure, element distribution, phase, and microhardness was studied.

The results demonstrate that the dendrites in the cladding layers decreased due to the external electromagnetic field, while the isometric and cellular-like crystals increased, thus realizing the effect of crystal refinement. Moreover, the element distribution in the transition area of the cladding layer was highly uniform, and the bonding strength between the cladding layer and the base increased. Given that the effects of the external electromagnetic field on temperature were significantly lower than those of the laser heat source, it influenced the phases slightly. The overall hardness of the cladding layer increased by 10–20% because of the external electromagnetic field. Therefore, laser cladding with electromagnetic field assistance improves the quality and performance of the cladding layer.

## Figures and Tables

**Table 1:** Chemical composition of AISI 1045 steel.

Elements	C	Si	Mn	Cr	Ni	Cu	Fe
wt %	0.45	0.20	0.50	0.24	0.22	0.25	Bal.

**Table 2:** Chemical compositions of Ni60AA.

Elements	C	Si	B	Cr	Fe	Ni
wt %	0.85	4.2	3.7	17	7.1	Bal.

**Table 3:** Orthogonal test table and scores.

No	*P* (W)	*v_0_* (mm/s)	*d_1_* (mm)	*L* (g/min)	Score
1	600	1	10	0.75	65
2	600	3	14	5.88	40
3	600	5	18	2.46	80
4	600	7	12	7.59	100
5	600	9	16	4.17	100
6	800	1	18	5.88	60
7	800	3	12	2.46	65
8	800	5	16	7.59	90
9	800	7	10	4.17	120
10	800	9	14	0.75	110
11	1000	1	16	2.46	40
12	1000	3	10	7.59	100
13	1000	5	14	4.17	100
14	1000	7	18	0.75	133
15	1000	9	12	5.88	110
16	1200	1	14	7.59	65
17	1200	3	18	4.17	75
18	1200	5	12	0.75	105
19	1200	7	16	5.88	115
20	1200	9	10	2.46	120
21	1400	1	12	4.17	25
22	1400	3	16	0.75	95
23	1400	5	10	5.88	125
24	1400	7	14	2.46	118
25	1400	9	18	7.59	135
k1	77	51	106	101.6	
k2	89	75	81	84.6	
k3	96.6	100	86.6	84	
k4	96	117.2	88	90	
k5	99.6	115	96.6	98	
R	22.6	66.2	25	17.6	
Optimal level	1400	7	10	0.75	
Order of significance	3	1	2	4	

**Table 4:** Process parameters.

No	Electromagnetic Field (Yes/No)	*d_1_* (mm)	*v_0_* (mm/s)	*P* (W)
1	No	12	5	1200
2	No	18	8	1800
3	Yes	12	5	1200
4	Yes	18	8	1800

**Table 5:** Process parameters.

Electromagnetic Field (Yes/No)	*d_1_* (mm)	*v_0_* (mm/s)	*P* (W)	*p_b_* (MPa)	*p_f_* (MPa)
No	18	8	1800	0.2	0.1

**Table 6:** Process parameters.

Electromagnetic Field (Yes/No)	*d_1_* (mm)	*v_0_* (mm/s)	*P*(W)	*p_b_* (MPa)	*p_f_* (MPa)
Yes	18	6	1200	0.2	0.1

## Data Availability

Data available on request from the authors.
